# Psychosis With Religious Delusions in a Reportedly Intersex Transgender Person

**DOI:** 10.7759/cureus.38192

**Published:** 2023-04-27

**Authors:** Allyson J Kemp, Mengpin Zhang, You Wang, Luba V Leontieva, Susan D Sperry

**Affiliations:** 1 Psychiatry, State University of New York Upstate Medical University, Syracuse, USA; 2 Psychology, State University of New York Upstate Medical University, Syracuse, USA

**Keywords:** gender dysphoria, intersex, transgender, religious delusions, psychosis

## Abstract

There is limited research on mental illness in intersex and transgender individuals. This case report describes psychosis in a self-identified intersex transgender individual with a past psychiatric history of schizoaffective disorder. The patient and collateral information reported colpocleisis as a newborn, was assigned and raised as a male, then transitioned to a female. When the patient discussed her experiences as a transgender person, she would become significantly more psychotic with disorganized speech and grandiose Christian delusions. A psychological assessment including a projective test was completed to better understand the patient’s psychotic symptoms along with her views of self, others, and the world. This case explores how the psychotic process interacts with gender dysphoria in a predominantly cisnormative, Christian society, with discussions of psychological defenses and psychodynamic theory.

## Introduction

This case study examines transgender identity and psychoticism with a religious preoccupation in a hospitalized patient. The patient self-identified as an intersex person, assigned male by doctors who later transitioned to female. The difficulty transgender individuals face in a cisnormative society while they explore their experiences with symptoms of psychosis and religious delusions is analyzed. Gender and religion create order and require conformity to an accepted societal norm. Religion can offer an escape from a harsh reality or result in rejection. Individuals who were raised in a religious environment may struggle to understand a self that does not conform to religious teachings. Psychosis is viewed by many experts as a defense to control an intolerable reality. As discussed below, while research is limited, psychosis is noted to be more often diagnosed in the transgender population.

About 0.5% of U.S. adults identify as transgender, and this figure increases to 1.4% in youth aged 13 to 17 [1]. Despite increasing proportions of individuals identifying as transgender, this population is not well-studied in mental health literature. A study of over 25,000 transgender individuals in inpatient psychiatric encounters found that 77% of encounters involving transgender individuals had at least one mental health diagnosis compared to 37.8% of hospital encounters involving cisgender individuals [2]. Psychosis was found to be 2.46 times more common in encounters involving transgender individuals compared to cisgender individuals [2]. Transgender individuals are three to 49.7 times more likely to be diagnosed with schizophrenia spectrum disorder than a cisgender person, which may reflect diagnostic biases and unique factors in the development of psychosis in transgender individuals [3]. Research on intersex individuals is even more limited. About 1.7% of people are born intersex and one in 2,000 babies (0.05% of humans) are born with genital differences that may result in recommendations for surgery [4]. A study on intersex individuals found that 53.6% reported their mental health as fair/poor, with the most common diagnoses being depressive disorders at 61.1%, anxiety disorders at 62.6%, and post-traumatic stress disorder (PTSD) at 40.9% [5].

Psychotic disorders such as schizophrenia have been referred to as “I am” illnesses, which pertain to the loss of individual identity and a sense of continual knowledge of self that is distinctive to a person [6]. For example, patients with schizophrenia tend to endorse statements, such as “I didn’t know who I was,” “I thought I had children,” “my sex changed,” or “my body or parts of it changed” [7]. This fluidity in self-image can give rise to a desire to change aspects of oneself, including appearance, gender, or religion [6]. It is important not to diagnose gender dysphoria solely during a psychotic episode given the intrinsic lack of stable identity that often defines psychosis; re-evaluation of gender dysphoria is recommended after psychotic symptoms are stabilized [8]. Transgender people may also self-describe as psychotic due to a lack of their own understanding of their own identities and experiences [3]. Historically, non-cisnormative gender identities have been linked to psychopathology and psychosis [3]. Psychotic diagnoses may be over-represented among individuals with non-cisnormative gender identities due to biases [3]. Transgender people are also noted to have higher rates of substance use increasing the chances for a psychotic disorder diagnosis [3].

A common theme in psychotic disorders is religious delusions, which exist in 15% to 39% of psychotic disorders [9]. Religious delusions are associated with overall poorer prognoses, including higher disability and distress, higher convictions in false beliefs, poor outpatient engagement, and persistent longer durations of untreated psychosis [9]. In non-psychotic individuals, religion has protective effects, such as being correlated with fewer depressive symptoms [10] and lower suicide rates [11]. However, religious identity can be a source of conflict due to rejection and ostracism for those with non-cisnormative gender identities [12]. Lesbian, gay, bisexual, transgender, queer (or questioning), intersex, and asexual (or allies, aromantic, or agender) (LGBTQIA) individuals being raised in religious households have been found to be at a higher risk for experiencing suicidal ideation and having a higher number of past suicide attempts compared to cisnormative individuals [12].

## Case presentation

Medical history

This case presents a 41-year-old, born of Native American heritage, Baptist religion, self-identified intersex person preferring female pronouns, with reported collateral and limited documentation for a past psychiatric history of schizoaffective disorder bipolar type, opioid use disorder, stimulant use disorder, gender dysphoria, and homelessness. Collateral and previous documentation painted a picture of an individual who had been chronically ill at varying intervals and poorly adherent to treatment. The patient was admitted for inability to care for self secondary to gross psychosis with no known onset for this specific psychotic episode, although psychosis was first noted in the patient’s 20s. There was no known reason for symptom decompensation at this time other than suspected medication non-compliance. On admission, the patient’s thought process, speech, and behaviors were disorganized. She was hyper-talkative and tangential with derailment and loose associations, clanging, thought blocking, and gesturing with her hands. She appeared to be internally preoccupied, responding to internal auditory hallucinations, with prominent grandiose Christian religious delusions. The patient was tried on risperidone 3 mg by mouth twice daily and ultimately treated with paliperidone long-acting injection (LAI) 234 mg and a booster paliperidone LAI 156 mg injection one week later after the initial injection in addition to oxcarbazepine 300 mg by mouth twice daily. After treatment, when given a semi-structured interview, the patient made occasional odd comments, but largely more coherent, linear, and less thought-disordered. Despite no reported trauma, the patient was verbally discriminated against and physically assaulted on the inpatient psychiatric unit by a peer who believed her to be homosexual; she was then transferred to a different psychiatric unit where she was physically assaulted a second time. Even after treatment, up to the day of discharge, the patient would become markedly religiously preoccupied, grandiose, and disorganized when discussing her family or sexuality in an unstructured manner. Topics of gender and sexuality were interlinked with family and religion. A psychological assessment was completed the day before discharge to specifically explore these phenomena in the context of this patient’s specific psyche.

Per the patient and collateral, her father chose colpocleisis/vaginal closure surgery under medical recommendation when the patient was a newborn. The patient was raised as a male and started to transition to a female in her teens; there is an unknown correlation between the patient’s sexual identity and the first presentation of psychosis in her 20s. The patient started hormones in her 20s but stopped them and decided to return to living as a man. She had a long history of multiple aliases, different used names, and fluctuating gender preferences. The patient’s reported biological hermaphroditism is difficult to confirm as hospital records older than within the last 10 years were destroyed, the patient’s parents were unavailable to confirm, and no diagnostic tests were performed. Furthermore, a true diagnosis of hermaphroditism requires the presence of both ovarian and testicular tissue in the gonad(s) diagnosed with ultrasound and histological study [13].

Psychological findings

After psychiatric stabilization, the psychology team conducted a clinical interview and administered the Thematic Apperception Test (TAT), a projective test in which the patient was asked to create stories in response to an image. The TAT offers a snapshot not only of patients’ thought processes and thought content but also of their views of self, others, and the world. Of interest, the patient’s presentation at the time of assessment, which occurred the day before discharge, was generally organized, but when asked to give a free unstructured narrative of her history, she became quite disorganized in speech and thought as well as delusional and religiously preoccupied. Throughout the interview, the patient was physically restless, pacing in the meeting room, and stepping out of the room two times during the one-hour meeting. Her affect was labile and reactive; her affect was full in range but was noted to be at times incongruent to topics discussed. Her speech was normal in rate and somewhat loud in volume, with occasional clanging, and at times she burst into song. Her thought process was markedly disorganized, non-linear, and referential, with derailment, loose associations, tangentiality, and evidence of thought blocking. She often responded to questions with unrelated answers, said bizarre and illogical things, and shifted topics frequently. Her thought content evidenced religious preoccupation. At times, she appeared to be responding to internal stimuli and addressed unseen others, and she appeared to have visual hallucinations, extending her hands to show us stigmata. Her insight and capacity for judgment were significantly impaired. She was cooperative throughout our session, provided both verbal and written consent, and repeatedly discussed throughout our interview her intent to help us and others by providing information.

In her clinical interview, the patient provided information about family members and important events in her life, and this information was interwoven with delusional material. Discussion of her gender identity was mixed with religious references. She spoke about being transgender, she also discussed being washed in the water of God and in his blood by being birthed by a woman. For example, she stated that she “was born a hermaphrodite, although I never speak it that way. I speak it that I am a transgender person.” She continued to say that “I feel that I am not because I know the truth about how transgendered give birth.” She explained, “I mean I was washed in the water of God and then washed in his blood by being born through the womb of a woman.” When directed back to speak about her family, she referred to her mother as virgin “Mary of Heaven,” and described how she was “hanging on a cross in the past.” She also at one point referred to herself by her first name followed by the word “Christ.” She described experiences of trauma, referring to being beaten by a woman when the patient was pregnant, explaining that when she said she was pregnant, she meant “pregnant with words.” She also told us that she was beaten by men and verbally abused with expletives accusing her of being homosexual. After describing this, she then began to gesture forcefully down toward the seat of a chair; when asked what she was doing, she replied, “That’s Jesus of Nazareth. I’m making him sit here and listen to myself.” She spoke in angry tones, saying, “Jesus, if you don’t listen to me from heaven, I know where you run to. Because he done it to me as a kid. He switched me back and forth.” She also claimed to be an important historical figure, working in counterterrorism for the government, and alluded to contact with aliens. She repeatedly told us that she was always a woman and repeatedly stated that she was telling the truth.

The patient was shown five cards from the TAT and her stories continued to be disorganized, non-linear, and bizarre, including references to gender identity, religion, and aliens. In response to the initial card, she told a story about a boy crying and praying over a fiddle and stated that the boy was thinking that all women should be able to give birth, however, they want to; she then referred to extraterrestrials and God and ended the story with the boy throwing the fiddle to his family in heaven. She said that the second card depicted a woman who was advising her daughter not to become a maid like her, then she digressed to talk about her physical attraction to women and her sense of being both a man and a woman. For the third card, she told a story about a man approaching a woman sexually, even though his wife was coming home soon; the man is upset when the woman asserts herself to deny him the right to touch her. When she viewed the fourth card, she told us, “This is one of your guardian angels, and she’ll sit on a woman’s shoulder … and she will whisper bad words to you.” She continued to tell us that she was the angel to whom the woman was listening and that she wanted to whisper only kind words to everyone. She also told us, “I’m the angel behind all angels.” Finally, in response to the blank card, she grew more disorganized and religiously preoccupied, stating this story would have three characters, explaining, “There’s the father, the son, and the holy ghost. No one knows if the holy ghost is a man or woman, right?” She continued by referencing aliens and told us that there was a fight over her, explaining, “The reason it’s happening is because I’m something that I can’t help being.” This patient’s stories echoed her personal struggles with both male and female aspects of self. She addressed issues of trauma and unwanted sexual advances. She related a sense of her personal power and uniqueness by aligning her identity with religious figures and claiming to have contact with extraterrestrials. Finally, she told stories of navigating good and evil and saw her own struggles through the lens of religious battles.

## Discussion

In this case, there was a connection between the triad of psychosis, the intersex transgender experience, and prominent religious delusions. There are many theories behind the equifinal association between increased diagnosis of psychosis in transgender persons. One suggested cause is the minority stress that transgender individuals experience in societies that prize cisnormative ideologies, leading to obvious external stigma but also internal stigma priming a person for a destabilized psyche and decompensation [3].

From a psychodynamic perspective, individuals with psychosis and transgender persons struggle to integrate self-experience and reality. Schizophrenia has been labeled as a disturbance of one’s core identity as there is no sense of self for the person [14]. The psychotic mind cannot differentiate between the self and emotions, ideas, words, experiences, and other people, all of which leads to a conglomerated mentation that cannot integrate conflicts nor problem-solve [15]. It is suggested that the patient’s psychosis developed when stress outweighed the mind’s abilities and reserves as a defense toward an intolerable reality to create a new space for self-preservation.

Transgender persons conform to their identified gender and sex rather than their assigned birth sex. However, most transgendered persons are living in a society that expects gender to match the biologically assigned sex, with a focus on reproduction ability. After transition, it is suggested that transgender persons, regardless of the degree of conformity, may struggle with continuous efforts to conform themselves to their identified gender and sex, not only to recognize their own self and to feel belongingness but also to avoid violence stemming from transphobia [16]. Unfortunately, as demonstrated in this case, histories of physical and sexual assault are common among transgender persons. It has been suggested that binary genders serve as a form of social order to promote reproduction; in the 1950s, gender was medicalized, specifically with intersex infants to justify sex reassignments, with the argued purpose to uphold social order [17]. Furthermore, intersex and transgender individuals destabilize the preferred simplified dichotomy of gender in the majority of societies. It has been pointed out that, in general, most societies hold a negative view toward transgendered persons.

Like gender, religion is a type of societal control of behavior to provide order. Religion offers personal and group acceptance and hope, as well as support and purpose outside of the self. While religion offers integration into a congregation, there is also a fear of rejection, in this case, for being transgender. Specifically, the more one identifies as religious with Christianity, the rates of transphobia and trans prejudice increase toward people deemed as social outgroups [18]. Religious delusions are common in schizophrenia with themes of self-significance including persecution and special abilities. In Christianity, women are given a special role as mothers, stemming from the motif of the Virgin Mary and Jesus, which the patient identified with. The patient discussed the womb, being pregnant, and a desire to be able to give birth, combined with religious delusions. When given a blank TAT card from the psychological assessment, a card that the patient interpreted as “who am I,” the patient discussed being both a male and a female. As the patient discussed being intersex and transgender, religious delusions not only increased but they morphed into increasingly fantastical delusions about extraterrestrials. The patient discussed being a creation of God, that Jesus switched them back and forth, and finally as an extraterrestrial. As the patient struggled to integrate self and identity, they clung to religious delusions, as religion offers the notion of acceptance but also places this person in an outgroup, describing themselves as an “extraterrestrial,” for being transgender.

## Conclusions

This case study examined psychoticism and religious delusions in a transgender individual with a self- and collateral-reported history of surgical sex assignment in infancy. Psychosis is prevalent in transgender persons, and the risk of a psychotic disorder diagnosis may be heightened by social stressors related to gender minority status (discrimination, marginalization, trauma experiences, and self-stigma), thus increasing vulnerability to psychosis. Increased psychotic disorder diagnoses may also be an overpathologized response to normative reactions against oppression. Increased rates of substance use among trans people, as was seen in our patient, may also contribute to high rates of psychosis. Religious delusions are common in psychosis, which may produce further psychological stress, as religion is often associated with transphobia. The patient was struggling to psychologically integrate a difficult reality in which gender and religion provide behaviors to fit cultural norms/societal expectations. The intersections between gender identity, society, and religion create a very stressful reality for transgender persons and these personal experiences and stressors influence and contribute to a trans person’s psychotic decompensation. The presentation of psychosis with religious delusions in this case, from a psychodynamic and psychiatric perspective, can be read as a possible attempt to tolerate a difficult stressful reality for transgendered persons. Religious delusions and psychotic symptoms may represent an attempt to integrate and control a sense of self while simultaneously holding a desire for acceptance and a fear of rejection.

As increasing numbers of individuals in the United States identify as non-binary or transgender, mental health providers are challenged to provide appropriate care that does not stigmatize and pathologize fluctuations in gender identity and expression. Increased attention and awareness of the impact of social and environmental stressors (e.g., discrimination, bullying, childhood trauma, exclusion from the majority) on individuals in the LGBTQIA community is essential to the provision of care. Integration of mental health services in primary care clinics providing gender-inclusive medical services may be an effective route to increasing access to mental health services. Further research is needed to better understand how transgender people can be best supported in their struggle to understand and integrate views of self.
